# Exploring cuproptosis as a mechanism and potential intervention target in cardiovascular diseases

**DOI:** 10.3389/fphar.2023.1229297

**Published:** 2023-08-11

**Authors:** Yang Yang, Qi Feng, Ying Luan, Hui Liu, Yuxue Jiao, Huijie Hao, Bo Yu, Yi Luan, Kaidi Ren

**Affiliations:** ^1^ Clinical Systems Biology Research Laboratories, Translational Medicine Center, The First Affiliated Hospital of Zhengzhou University, Zhengzhou, China; ^2^ Research Institute of Nephrology, The First Affiliated Hospital of Zhengzhou University, Zhengzhou, China; ^3^ Department of Integrated Traditional and Western Nephrology, The First Affiliated Hospital of Zhengzhou University, Zhengzhou, China; ^4^ State Key Laboratory for Artificial Microstructures and Mesoscopic Physics, School of Physics, Peking University, Beijing, China; ^5^ School of Laboratory Medicine, Xinxiang Medical University, Xinxiang, Henan, China; ^6^ Tianjin Key Laboratory of Retinal Functions and Diseases, Tianjin Branch of National Clinical Research Center for Ocular Disease, Eye Institute and School of Optometry, Tianjin Medical University Eye Hospital, Tianjin, China; ^7^ Department of Pharmacy, The First Affiliated Hospital of Zhengzhou University, Zhengzhou, China; ^8^ Henan Key Laboratory of Precision Clinical Pharmacy, Zhengzhou University, Zhengzhou, China

**Keywords:** copper (Cu), cuproptosis, cardiomyocytes, cardiovascular disease (CVD), intervention targets

## Abstract

Copper (Cu) is a vital trace element for maintaining human health. Current evidence suggests that genes responsible for regulating copper influx and detoxification help preserve its homeostasis. Adequate Cu levels sustain normal cardiac and blood vessel activity by maintaining mitochondrial function. Cuproptosis, unlike other forms of cell death, is characterized by alterations in mitochondrial enzymes. Therapeutics targeting cuproptosis in cardiovascular diseases (CVDs) mainly include copper chelators, inhibitors of copper chaperone proteins, and copper ionophores. In this review, we expound on the primary mechanisms, critical proteins, and signaling pathways involved in cuproptosis, along with its impact on CVDs and the role it plays in different types of cells. Additionally, we explored the influence of key regulatory proteins and signaling pathways associated with cuproptosis on CVDs and determined whether intervening in copper metabolism and cuproptosis can enhance the outcomes of CVDs. The insights from this review provide a fresh perspective on the pathogenesis of CVDs and new targets for intervention in these diseases.

## Introduction

Copper (Cu) is an essential trace element for human health and is the third most abundant element in humans (Bost et al., 2016). Copper is mainly derived from foods such as vegetables, shellfish, seeds and nuts, and organ meats. The average daily copper intake ranges from 1 to 1.6 mg, and the recommendation for adults is 0.9 mg/day. The copper concentration in the body ranges from 50 mg to 120 mg and is mainly distributed in the brain, liver, and bones, while it is less abundant in the heart and kidney (Zeinali et al., 2019). The absorption of copper relies on the proximal small intestine and stomach with the aid of the acidic environment, which can degrade dietary macromolecules to release copper (van den Berghe and Klomp, 2009). Where copper intake is inadequate, active transport mechanisms facilitate its absorption. Conversely, passive diffusion mechanisms aid its absorption when copper intake is high. Once ingested, copper is delivered to plasma amino acids and albumin before being transferred to the liver. Subsequently, copper binds to ceruloplasmin in the liver and is distributed to peripheral tissues. Copper can also be stored in the liver with the aid of metallothionein.

Copper catalyzes reactions in a variety of physiological processes, including mitochondrial energy production, tyrosine and neurotransmitter metabolism, redox homeostasis, and extracellular matrix remodeling. Copper maintains normal hematopoietic function and is also involved in iron metabolism and erythropoiesis (Takahashi, 2022). Copper exists in two oxidative states, Cu(I)/Cu^+^ (cuprous ion) and Cu(II)/Cu^2+^ (cupric ion). Cu^+^ preferentially binds to cysteine with the thiol group or methionine with the thioether group. In contrast, Cu^2+^ has a high affinity for the imidazole nitrogen group in histidine or the 2nd carboxyl group of aspartic/glutamic acid (Chen et al., 2020). The copper ions can easily form macromolecules with these amino acids. In addition, copper is involved in a multitude of proteins, such as cytochrome c oxidase (CcO), copper/zinc superoxide dismutase [Cu/Zn superoxide dismutase (SOD)], cAMP-degrading phosphodiesterase PDE3B, and mitogen-activated protein kinase MEK1 (Harrison and Dameron, 1999). Among these, copper mediates various biochemical reactions by serving as an electron donor or receiver and maintains specific protein structures by incorporating these proteins (Ruiz et al., 2021).

Copper acts as a key catalytic cofactor in biological processes. The intracellular copper concentration is kept in a relatively low range, and a moderate increase will cause cytotoxicity and even lead to cell death. Therefore, the absorption, distribution and elimination of copper are strictly regulated (Chen et al., 2022). In humans, genetic mutations that lead to copper accumulation have been linked to serious, potentially life-threatening pathological conditions and diseases such as CVDs (Chen et al., 2022).

Although copper is less abundant than iron in many organisms, it is essential for many biological processes, such as mitochondrial respiration. The oxidative state switch of copper induces the generation of reactive oxygen species (ROS), which damage lipids, nucleic acids, and proteins and can also block the synthesis of iron-sulfur clusters (Cui et al., 2022). Given the vital role of copper in organisms, the precise regulation of copper is crucial for maintaining the homeostasis of living organisms.

### Copper metabolism in mammals

Copper undergoes uptake, transport, and exit in multicellular animals, and several molecules orchestrate these processes. These processes are discussed below.

#### Uptake of copper ions

Copper intake primarily occurs through dietary sources, and most adults can obtain sufficient copper from daily diets. However, pregnant women as well as infants require more copper intake. In the digestive tract, copper is digested by enterocytes in the form of Cu^2+^, which is the optimal form for absorption. Cu^2+^ uptake is directly mediated by divalent metal transporter 1 (DMT1) (Jiang et al., 2013). Cu^2+^ cannot be directly used by cells and can be reduced into Cu^+^ in all cell types by 4 reductases: Duodenal cytochromeb (DCYTB) and 6 transmembrane epithelial antigen of the prostate (STEAP) 2, 3, and 4. Cu^+^ ions are taken up by copper transporter 1 (Ctr1) via a high-affinity mechanism. It is widely recognized that Ctr1-mediated Cu^+^ uptake is the primary method of incorporating cupric iron into peripheral tissues (Cui et al., 2022). The incorporation process in the enterocytes is more complex.

The location of Ctr1 at the apical membrane of enterocytes suggests that Ctr1-mediated dietary copper uptake occurs in the intestinal lumen (Nose et al., 2006). Contrasting studies revealed that Ctr1 is also located in basolateral membranes and inside enterocytes. In addition, copper intake from the intestinal lumen is not dependent on Ctr1, as inactivation of Ctr1 has little impact on copper accumulation in enterocytes (van den Berghe and Klomp, 2009). These results suggest that copper translocation across the apical membrane may be mediated by other transporters, such as DMT1, or by endocytosis. It is highly conceivable that Ctr1 plays an essential role in dietary copper utilization through hitherto undocumented mechanisms. When Ctr1 is disrupted, copper uptake by other processes cannot be utilized in the organism, as it is sequestered in subapical vesicles. This has been confirmed by a study in mice that demonstrated that Ctr1 ablation resulted in severe copper deficiency in peripheral tissues, which was rescued by intraperitoneal injection of copper (Nose et al., 2006).

#### Distribution of copper ions

After uptake, some copper ions can be transferred to cuproproteins (CuPrs) by 3 pathways: Cytosolic, Golgi and mitochondrial (Chen et al., 2020). In the cytosol, copper chaperone for SOD1 (CCS) promotes Cu^+^ loading and SOD1 activation. *CCS* knockout mice verified the role of CCS and SOD1 in ROS scavenging in germ line cells and neurons (Wong et al., 2000). Recent studies suggest that copper is obtained by CCS from Ctr1 and transferred to SOD1 by forming a Ctr1-CCS-SOD1 complex. This complex can be disrupted by the full activation of SOD1. Apart from CCS, the chaperones cytochrome C oxidase copper chaperone (COX17) and antioxidant-1 (ATOX1) can also transfer Cu^+^ from Ctr1 (Matson Dzebo et al., 2016). Cuprous ions are transferred to cytochrome c oxidase 1 (SCO1) and SCO2 by COX17, located at the mitochondrial inner membrane. SCO1 and SCO2 then deliver copper to COX2. Additionally, COX17 can deliver copper to COX11 from the cytoplasm, which is then transferred to COX1 (Matson Dzebo et al., 2016). Both COX1 and COX2, as subunits of CcO, are required for the oxidative phosphorylation process. Moreover, COX17 is believed to be crucial for CcO biogenesis.

**FIGURE 1:**
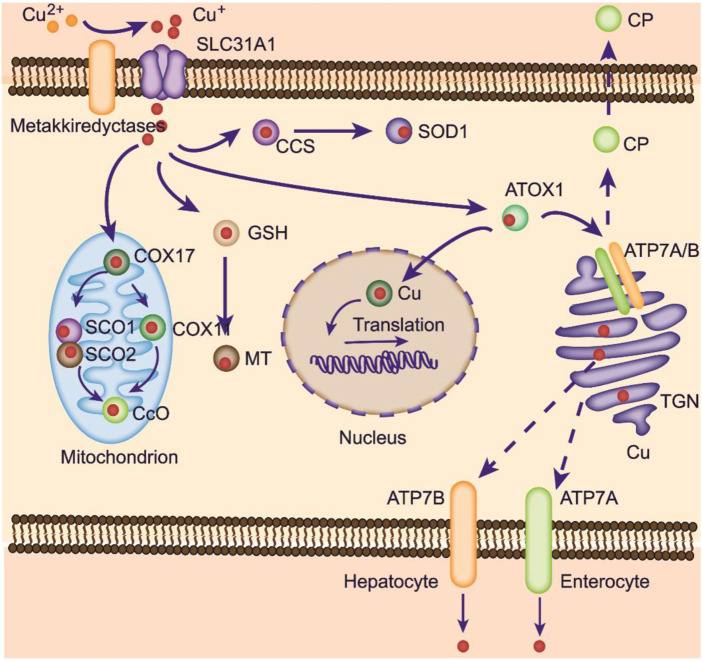
Copper metabolism within cells. Copper can be transported to mitochondria for storage. At physiological copper levels, copper-transporting ATPases located on the TGN deliver Cu from the cytoplasm into the TGN. With intracellular Cu^+^ accumulation, these ATPases translocate to the plasma membrane to export Cu^+^. ATP7A is responsible for copper pumping into the liver in the basolateral membrane of enterocytes. Excess copper is exported into the bile via ATP7B. ATOX1 mediates copper delivery to the nucleus, where copper binds to transcription factors and promotes gene expression. COX17 is crucial in transporting copper to SCO1, SCO2, and COX11, facilitating its delivery to CcO to activate the enzymes involved in the respiratory chain.

ATOX1 delivers copper to ATPase copper transporting *α* (ATP7A) and *ß* (ATP7B) (Muller and Klomp, 2009) (Figure 1). These ATPase locations vary from the trans-Golgi network (TGN) to the plasma membrane under different circumstances. Plasma membrane-localized ATPases are important for the systemic distribution of copper ions, while other TGN membrane-localized transporters are responsible for copper loading to various CuPrs, such as ceruloplasmin (CP) and SOD3. Afterward, these copper-bound CuPrs are targeted to specific organelles or secreted into the extracellular matrix.

In enterocytes, absorbed copper ions are transported to the circulatory system for delivery to other tissues, a process that relies on ATOX1/ATPase (Takahashi, 2022). Defects in the ATOX1/ATP7A process lead to damaged copper distribution, causing pathological alterations in several organs (Kaler, 2011). Copper ions in the blood can bind to albumins and free amino acids and are further taken up by Ctr1 in peripheral organs. The liver is a pivotal organ for copper storage. Excess copper ions in hepatocytes bind to cytosolic metallothioneins (MTs) or accumulate within the lysosome as a pool. A large amount of copper ions is delivered to the CP by the ATOX1/ATP7B/Golgi route (Figure 1) (Blockhuys et al., 2020). Then, CP is transferred to the blood, oxidizing Fe^2+^ into Fe^3+^ to promote its absorption.

#### Excretion of copper ions

Copper ions can be exported from cells by a series of pathways. CuPrs loaded with Cu^+^ can be secreted out of cells. In enterocytes, absorbed Cu^+^ is directly secreted into the blood with the help of ATP7A at the basolateral membrane, transferred to the liver, and incorporated by hepatocytes in a Ctr1-dependent manner (Figure 1). Within hepatocytes, CP is synthesized and loaded with Cu^+^ at the TGN and then secreted into blood. Upon excess copper levels, hepatic ATP7B translocates from the Golgi apparatus to the lysosome and moves copper to its lumen (Petruzzelli et al., 2022). Then, ATP7B promotes exocytosis mediated by p62, importing excess copper into the bile and subsequently exiled out for the body (Polishchuk et al., 2014). The gastrointestinal tract can also influence the direct transport of copper by hepatic ATP7B on the apical membrane.

### Copper balance in mammals

In mammalian cells, copper homeostasis is maintained by regulating genes related to copper influx and detoxification (Bertinato and L’Abbe, 2004). The level of Ctr1 is negatively modulated by intracellular copper levels (Petris et al., 2003). When copper levels are high, Ctr1 is internalized from the cell surface as a response. Conversely, when copper levels are low, internalized copper can be released back into the plasma membrane. This regulatory mechanism can account for the varying efficiency of copper incorporation in enterocytes in response to different dietary copper doses. Moreover, high cellular copper ion levels can enhance the expression of metallothionein genes to remove excess and toxic copper ions (Ge et al., 2022). Under low cellular copper ions, nonion-bound MTs are easily degraded, leading to a low level of these chelators.

To date, a multitude of studies have revealed that mammals have evolved a sophisticated system to detect and regulate copper levels in various organs, although the underlying mechanisms of this system are not yet fully understood. Earlier studies used two naturally occurring copper isotopes (63Cu and 65Cu) for copper turnover measurement. Meanwhile, copper conservation in different organs can be highly specific: copper conservation in brains and hearts is very efficient, with little copper loss, while in the liver, copper conservation is induced only under significant copper loss conditions (Levenson, 1998).

### Copper functions within cells

Copper is an important regulator of numerous enzymes and is involved in several physiological processes, such as angiogenesis and neuromodulation. It is now understood that the P-type Wilson ATPase plays a critical role in blocking copper deficiency or toxicity and transfers copper from the liver to the secretory pathway (Dyla et al., 2020). Its mutation can induce a shortage in copper transport to the bile and defective incorporation of copper into ceruloplasmin. Other copper-incorporating enzymes also play important roles in processes, including oxidative processes, neurotransmitter synthesis, and bone formation. For instance, zinc-Cu superoxide dismutase is critical in oxidative processes (Tainer et al., 1983).

Under normal circumstances, the metabolic processes that generate ROS and processes that produce antioxidant agents are balanced. However, if this balance is disrupted, the levels of ROS in the circulatory system increase, leading to damage to various cellular structures. Ultimately, this damage is associated with the progression of chronic or degenerative diseases, such as cancer and cardiovascular diseases. Moreover, slight copper deficiency may be involved in the progression of several diseases, such as diabetes and cardiovascular diseases (CVDs) (Saari and Schuschke, 1999). In contrast, excessive copper levels can lead to detrimental effects on multiple organs, which can cause various diseases and even death.

### Cuproptosis is different from other known cell death modes

Current evidence suggests that excess trace elements may induce cell death via specific pathways (Ke et al., 2023). Ferroptosis is an iron-dependent form of cell death in the membrane triggered by excess iron (Fang et al., 2019). Apart from iron metabolic pathways, there are also other pathways involved in ferroptosis, including the cysteine/glutathione/glutathione peroxidase 4 (GSH/GPX4) axis, the guanosine triphosphate cyclohydrolase 1/tetrahydrobiopterin (GCH1/BH4) axis, and the ferroptosis suppressor protein 1/coenzyme Q (FSP1/CoQ) axis (Jiang et al., 2021; Qin et al., 2021).

Ferroptosis is implicated in a broad spectrum of human diseases, such as neurodegenerative diseases, kidney diseases, liver fibrosis, and cardiovascular disease (Tang et al., 2021; Liu et al., 2022; Zhang et al., 2022). In comparison with ferroptosis, cuproptosis remains poorly characterized. The mechanism of toxic action of copper on cells is different from other known regulatory cell death mechanisms (such as ferroptosis, etc.), and it is a new cell death mode, and it is named cuproptosis. Similar to ferroptosis, copper induces the aggregation of fatty acylated proteins and the instability of iron-sulfur tufting proteins by directly binding to the fatty acylated part of the tricarboxylic acid cycle, leading to protein toxic stress and thus inducing a mode of death independent of the apoptotic pathway, i.e., cuproptosis (Xiong et al., 2023). Both ferroptosis and cuproptosis are associated with mitochondria (Song et al., 2023).

**FIGURE 2:**
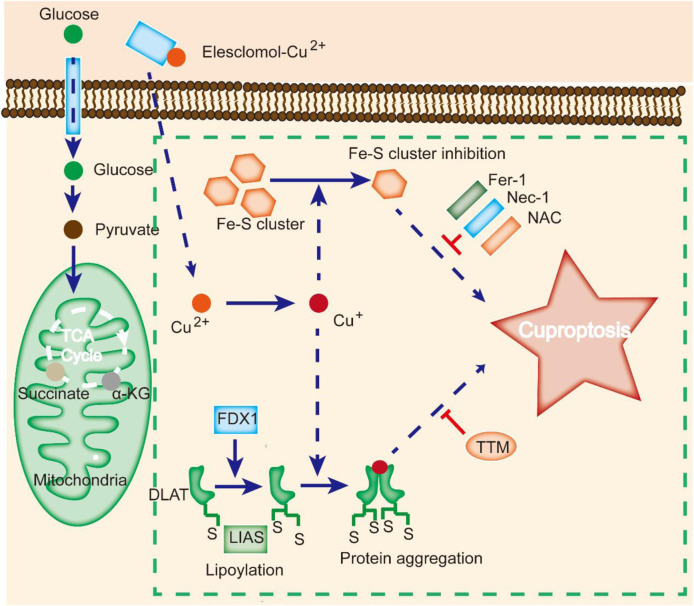
Cuproptosis is different from other cell death modes. Cu ionophores, such as elesclomol, bind Cu and transfer it to cells, where it binds to lipoylated enzymes of the TCA cycle. FDX1/LIAS modulates protein lipoylation, inducing protein aggregation and Fe-S cluster inhibition. Aberrations in these processes result in proteotoxic stress and ultimately cell death. Cu chelators inhibit cuproptosis, and inhibitors of other cell death modes, such as Fer-1 and Nec-1, do not affect cuproptosis.

Contrasting studies revealed that excessive Cu^2+^ might induce cell death by apoptosis, caspase-independent cell death, or ROS accumulation (Tang et al., 2022). A new type of regulated cell death known as cuproptosis was identified by Tsvetkov et al. as a result of intracellular copper accumulation. Cuproptosis is characterized by the aggregation of lipoylated mitochondrial enzymes and the loss of iron-sulfate (Fe-S) proteins. It was also demonstrated that the addition of Elesclomol, a Cu ionophore, at a concentration as low as 40 nM could raise the intracellular Cu level and trigger cuproptosis (Figure 2).

Notably, suppression of known cell death modes, such as necroptosis (necrostatin-1), apoptosis (Z-VAD-FMK), oxidative stress (N-acetyl cysteine), and ferroptosis (ferrostatin-1), all failed to inhibit elesclomol-induced cell death (Cobine and Brady, 2022). In contrast, treatment with a Cu chelator effectively suppressed excess Cu^2+^-induced cell death, indicating that cuproptosis is different from other types of cell death (Figure 2).

Cuproptosis is associated with changes in mitochondrial enzymes (Tang et al., 2022). Mitochondria are a key target in cuproptosis, with oxidative damage in the membrane and alterations in TCA cycle enzymes (Wang et al., 2023). Upon Cu overload in patients, CcO activity is reduced, and the TCA cycle is suppressed, leading to aconitase inactivation (Shen et al., 2022). A study revealed that cells treated with Cu ionophores showed dysregulation of several TCA cycle-related metabolites in a time-dependent manner, and inhibition of electron transport chain complexes I and II dramatically abrogated Cu-induced cell death (Li et al., 2022a). Meanwhile, metabolic enzymes can be altered by lipoylation, a highly conserved type of posttranslational modification (PTM) (Rowland et al., 2018). This modification is rare within mammalian cells and is completely enriched in the TCA cycle. For instance, dihydrolipoamide S-acetyltransferase (DLAT) is a lipoylated protein (Tajima et al., 2019). Cu can bind to DLAT, leading to the aggregation of lipoylated DLAT. Mitochondrial ferredoxin (FDX1) and lipoyl synthase (LIAS) have been reported to be critical regulators of Cu toxicity by genome-wide CRISPR screening (Figure 2) (Dreishpoon et al., 2023). Genetic ablation of FDX1 or LIAS contributes to the accumulation of α-ketoglutarate and pyruvate, reduction of lipoylation, and reduced cell death (Dreishpoon et al., 2023).

It is widely thought that Cu toxicity is also related to disrupted Fe-S-containing enzymes (Campos et al., 2021). The Fe-S pool in mitochondrial ferredoxin can be damaged by Cu, resulting in downstream growth inhibition. Additionally, Cu can block Fe-S cluster formation by suppressing the activity of mitochondrial assembly proteins involved in the process (Boniecki et al., 2017). Moreover, deficiency in the mitochondrial ABC transporter ATM1, a transporter for intermediates in Fe-S cluster formation, results in aggravated Cu-induced toxicity (Do et al., 2018). Since most Fe-S-containing proteins constitute cofactors for enzymes in the electron transport chain and other biological processes, mitochondrial protein aggregation may induce dysregulation of these Fe-S clusters and cell death.

### Cu function in CVDs

#### Cu function in atherosclerosis

Cu is reportedly essential in the pathogenesis of atherosclerosis (Chen et al., 2023). As indicated by epidemiological data, Cu levels are positively associated with the incidence of atherosclerosis (Rachinskii, 1967; Volker et al., 1997). Additionally, an increased concentration of copper has been observed in atherosclerotic plaques. The release of free copper ions is thought to induce neointima thickening and the development of arteriosclerotic lesions in damaged rat carotid arteries (Iskra et al., 1997). The progression of atherosclerotic lesions, neointima formation, and vascular inflammation were effectively inhibited by treatment with a Cu chelator in injured *ApoE* knockout mice (Lamb et al., 2005; Gao et al., 2018). Furthermore, in neointima vascular smooth muscle cells (VSMCs) or injured atherosclerotic vessels, the Cu chaperone ATOX1 and exporter ATP7A were found to be highly accumulated and colocalized (Lamb et al., 2005; Wei et al., 2012). In addition, ATOX1 depletion in mice inhibited the expansion of the extracellular matrix (Habas and Shang, 2018).

The mechanism underlying Cu-mediated atherosclerosis remains largely unknown and may be attributed to the proinflammatory response of Cu in the progression of atherosclerosis (Wei et al., 2012). Cu deficiency inhibits the expression of adhesion molecules, activating endothelial cells by inducing the adhesion of leukocytes (Habas and Shang, 2018). Additionally, Cu deficiency promotes the level of cholesterol, a key risk factor in atherosclerosis (Malek et al., 2000; Ozumi et al., 2012). Cu deficiency may reduce NO levels via SOD1, leading to atherosclerosis via impaired endothelial function (Jeney et al., 2005; Bakavayev et al., 2019). Effective regulation of copper concentration is crucial in atherosclerosis and associated cardiovascular diseases due to the harmful effects of copper accumulation and deficiency on vascular function and integrity.

#### Cu in cardiac hypertrophy

Given that adequate Cu levels are essential for normal cardiac function by maintaining mitochondrial function (Mao et al., 1998; Zheng et al., 2015), Cu deficiency might lead to hypertrophic cardiomyopathy. Mutations in the synthesis of SCO2, a chaperone involved in trafficking Cu to cytochrome c oxidase, are detected in human cardiomyopathy. Cu deficiency may alter cardiac morphology, mitochondrial swelling, fragmentation, and enlarged myocytes (Medeiros et al., 1991). Moreover, Cu deficiency in the heart is accompanied by defective mitochondrial respiratory function and electrocardiographic abnormalities (Klevay, 2000). It has been established that Cu deficiency could also change the gene expression profile related to cardiac contractility, fibrosis, and calcium cycling (Elsherif et al., 2004; Stockwell et al., 2017). In the heart, an adequate Cu supply could restore many of the adverse effects of Cu depletion (Zhong et al., 2022). In addition, studies have shown that Cu depletion in mice resulted in lipid accumulation in myocardial tissues, accompanied by cardiac hypertrophy and mitochondrial swelling (Jiang et al., 2007; Yan et al., 2022). SCO2 mutation patients exhibited severe hypertrophic cardiomyopathy, and Cu-histidine treatment ameliorated heart function and normalized blood pressure (Jaksch et al., 2000).

#### Cu in ischemia/reperfusion injury

Tissue ischemia causes increased ROS and decreased NO in activated endothelial cells, which results in increased inflammatory factors such as interleukins and free radicals (Mittal et al., 2014). Relatively lower Cu could exacerbate the inflammatory response upon ischemia/reperfusion injury, whereas supplementation with Cu could ameliorate tissue damage during this process (Tural et al., 2020). The provision of bivalent copper ions resulted in a notable reduction in malondialdehyde and myeloperoxidase activity and increased glutathione content and SOD activity in rats, indicating a protective effect against tissue damage (Wada et al., 1994; Jaksch et al., 2000; Tanaka et al., 2004; Huang et al., 2011). Mitragynine, an anti-ischemia agent, was found to increase the levels of hepatic Cu/Zn-SOD and protect against liver inflammation and damage in ischemia/reperfusion injury (Hirao et al., 2022). This observation demonstrated the protective role of Cu in ischemia/reperfusion injury. Additionally, a recent human survey depicted that dietary copper intake negatively correlated with myocardial infarction (Wen et al., 2022).

In summary, an appropriate concentration of divalent copper ions can improve tissue damage caused by ischemia/reperfusion, while excessive copper levels may cause cell death (Blades et al., 2021). However, there is currently no consensus on the optimal concentration of copper for mitigating ischemia/reperfusion damage, possibly due to variations in the method of copper delivery.

Since dysregulation in Cu levels is associated with multiple diseases, its homeostasis should be tightly maintained (Kim et al., 2010). Further research is necessary to elucidate the precise mechanisms by which Cu dysregulation induces cellular damage, which could provide new strategies for preventing diseases or slowing their progression.

### Mechanism of cuproptosis in CVDs

#### Oxidative stress

**FIGURE 3:**
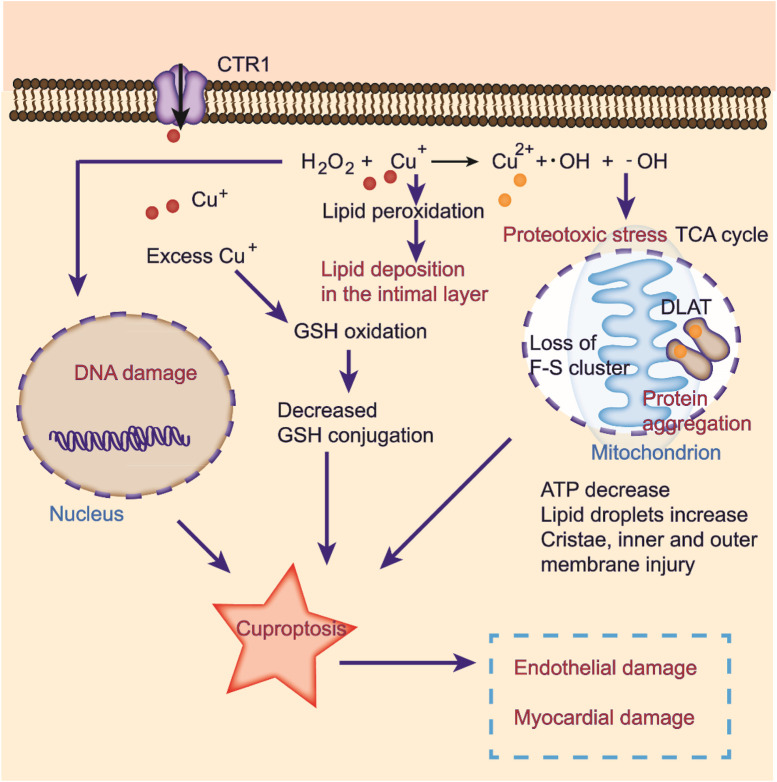
Cuproptosis induced by oxidative stress in CVDs. Excessive copper induces oxidative stress, elevating abnormal lipid metabolism and DNA damage by the Fenton reaction. Copper ions directly bind to fatty acylation components in the TCA cycle, inducing protein aggregation and dysfunction, inhibiting the TCA cycle, triggering proteotoxic stress, and ultimately cell death. Cuproptosis may lead to endothelial and myocardial damage.

Cells maintain a delicate balance between oxidation and antioxidation processes to prevent oxidative stress, cellular damage, and the onset of cardiovascular diseases (Forman and Zhang, 2021). Copper ions are involved in this cycle as they transition between oxidative and reductive states, which can lead to the formation of hydroxyl radicals that damage DNA and lipids (Figure 3) (Ruiz et al., 2021). Additionally, excess copper can disturb lipid metabolism and cause lipid accumulation in the intima layer, which contributes to atherosclerosis (Figure 3) (Blades et al., 2021). Copper exposure reportedly affects metabolite levels, especially in glycerophospholipid metabolism and fatty acid degradation and extension (Ruiz et al., 2021). Furthermore, oxidative stress induced by excess copper can lead to glutathione oxidation and reduced glutathione conjugation, ultimately resulting in cardiotoxicity (Figure 3) (Alqarni et al., 2019). Recent studies have identified copper-related proteotoxic stress as a potential factor in the pathogenesis of CVDs (Chen et al., 2023). Further research is needed to elucidate the mechanisms underlying copper-induced cellular damage and develop strategies for preventing or slowing the progression of related diseases.

#### Mitochondria and copper in CVDs

In the heart, mitochondria coordinate many metabolic processes (Luan et al., 2021a; Luan et al., 2021b; Gan, 2021; Ruiz et al., 2021). Copper serves as a critical factor in mitochondrial function (Mao et al., 1998; Ruiz et al., 2021). In mitochondria, CcO, a component of complex IV, activates the enzymes in the respiratory chain (Yin et al., 2018). Copper deficiency limits copper transport to SCO1/SCO2 and COX11 via COX17, reducing CcO synthesis (Ramchandani et al., 2021). Additionally, copper deficiency can induce mitochondrial damage by enhancing the expression of other mitochondrial proteins. Peroxisome proliferator-activated receptor-gamma coactivator-1 alpha protein (PGC-1α) can disrupt the structure of mitochondria, blocking mitochondrial proliferation and contributing to the development of myocardial diseases (Onishi et al., 2014). Elevated PGC-1α levels induced by copper deficiency have been shown to result in myocardial defects (Oliveri et al., 2015). In addition, reducing CcO activity leads to rigid cardiomyocyte fibers, ultimately contributing to severe cardiac disorders (Chen et al., 2023).

The mitochondrial respiration pathway is a necessary condition for cell death induced by copper ionophores (Tang et al., 2022). Cuproptosis is closely related to mitochondrial respiration. Excess intracellular copper can be transported to mitochondria via ionophores and directly bind to the fatty acylated components in the tricarboxylic acid cycle of mitochondrial respiration, resulting in the accumulation of fatty acylated proteins and the loss of iron sulfur tuftin, inducing protein toxic stress and eventually leading to cell death (Tsvetkov et al., 2022).

### Therapeutic approaches targeting cuproptosis in CVDs

#### Copper chelators

Therapeutics targeting cuproptosis in CVD mainly include copper chelators, inhibitors of copper chaperone proteins, and copper ionophores (Table 1) (Tang et al., 2022). Tetrathiomolybdate (TTM) is a copper-chelating agent that shows a high affinity for copper and is currently employed in treating Wilson’s disease, a condition characterized by the excessive accumulation of copper in the liver (Banfi et al., 2007). TTM achieves copper chelation by forming a specific complex of TTM-copper-ATX1 (Pufahl et al., 1997; Alvarez et al., 2010). This complex effectively inhibits copper delivery and subsequent incorporation into cuproproteins. TTM suppresses atherosclerosis in ApoE-deficient mice by lowering copper bioavailability (Wei et al., 2012). Triethylenetetramine (TETA) specifically binds to Cu^2+^ ions and is used as a second-line treatment for Wilson’s disease (Zhao et al., 2020). TETA blocks the elevation of serum copper and abolishes increased CP activity after myocardial ischemia injury (Yang et al., 2021). It has been demonstrated that TETA treatment could restore myocardial function in the hearts of diabetic patients by improving mitochondrial protein CcO, SOD1, and mt-CCS activity (Chen et al., 2023).

**TABLE 1:** Potential therapies targeting copper in cuproptosis-related CVDs.

Category	Copper drug	Function
Copper chelators (decrease the intracellular copper concentration)	Tetrathiomolybdate	Currently used in treating Wilson’s disease
	Triethylenetetramine	Second-line approach for Wilson’s disease. Restore myocardial function in the hearts of diabetic patients
	EDTA	Reduce recurrent cardiovascular events in type 1 and 2 diabetic patients with previous myocardial infarction
	Trientine dihydrochloride	Improve mitochondrial function in patients with hypertrophic cardiomyopathy
Natural antidote (decreases the intracellular copper concentration)	Turmeric	Widely used in metabolic diseases and CVDs
	Chalkophomycin	Is expected to have therapeutic effects on Wilson’s disease or CVDs
Inhibitor of copper chaperones (reduces the intracellular copper concentration, more specifically)	DCAC50	Specifically, inhibit cancer cell proliferation
Copper ionophore (transports copper into cells)	8-hydroxyquinoline	Chelate metals and exert a wide range of different diseases, such as neurodegenerative disease and cancer
	Compound 5b	Suppress metal (Cu^2+^ and Zn^2+^)-induced Aβ aggregation
	Elesclomol	Is used in cancer cells clinically
Targeted iron carrier-based metal supplements	N-acetylgalactosamine	More effective in transporting copper to the liver

The copper carrier 8-hydroxyquinoline and its derivatives can also chelate metals and exert a wide range of diseases, such as neurodegenerative disease and cancer (Chen et al., 2023). Compound 5b, a derivative of 8-hydroxyquinoline, has been reported to suppress metal (Cu^2+^ and Zn^2+^)-induced Aβ aggregation (Oliveri et al., 2015).

It is well established that disodium ethylene diamine tetraacetic acid (EDTA) can chelate many metals, including copper (Banfi et al., 2007). Interestingly, EDTA disodium infusion has been reported to reduce recurrent cardiovascular events in type 1 and 2 diabetic patients with previous myocardial infarction, as investigated by a 10-year clinical trial (National Heart et al., 2013). Trientine dihydrochloride is a copper chelator approved for Wilson’s disease treatment (Cooper, 2011). Trientine was demonstrated to selectively chelate Cu and improve mitochondrial function in patients with hypertrophic cardiomyopathy (Reid et al., 2022). Additionally, trientine restored mitochondrial structural damage and myocardial metabolic enzyme activity in diabetic patients (Ramli et al., 2022).

Metal chelators have several disadvantages, including heavy metal redistribution from other tissues to the brain, which can worsen brain toxicity and result in the loss of essential metals and liver toxicity (Yuan et al., 2017). However, natural antidotes have several advantages and are readily available. Turmeric, derived from the rhizome of the herb Curcuma longa, is an excellent metal chelator, including copper, due to its major ingredient curcumin, which has antioxidant, anti-inflammatory, and antiviral properties and is widely used in metabolic diseases and CVDs (Su et al., 2019; Guo et al., 2023). The effectiveness of turmeric in the treatment of CVDs has been demonstrated. Methanobactin, a copper chelator, holds promise for treating copper overload-induced diseases (Zheng et al., 2022). Chalkophomycin, a copper chelator recently discovered by Gong et al., is anticipated to have therapeutic benefits for Wilson’s disease, neurodegenerative diseases, or CVDs [106]. Since CVDs require long-term cooperative treatment, future studies in this area are urgently needed.

#### Small-molecule inhibitors of copper chaperone proteins

Copper ion chelators often lead to low copper concentrations, which have many side effects and disrupt many phycological processes that require copper (Chen et al., 2023). Thus, approaches that selectively reduce copper levels and redistribute copper ions are essential for therapeutic effects while maintaining low side effects. It has been reported that the compound DCAC50 could specifically inhibit cancer cell proliferation while not interfering with normal cells by limiting intracellular copper ion delivery and binding to the copper chaperone proteins ATOX1 and CCS (Karginova et al., 2019). DCAC50 exerts its effects by inhibiting the activity of Cu/Zn SOD1, which binds to copper ions and regulates copper transport within cells. Inhibition of Cu/Zn SOD1 activity by DCAC50 leads to increased ROS levels, damaging mitochondrial functions and impairing cellular homeostasis (Leitch et al., 2009). Moreover, DCAC50 sensitizes cancer cells to chemotherapy (Karginova et al., 2019).

A study revealed that *ApoE* ^−/−^ mice with atherosclerotic lesions exhibited upregulation of ATOX1 in the intima (Ozumi et al., 2012). In inflammatory endothelial cells, the binding of ATOX1 to TNF-α receptor-associated factor 4 (TRAF4) induces ROS production dependent on Cu (Das et al., 2019). The ATOX1-TRAF4 pathway may serve as a potential target for vascular diseases. Cu is transported to the major cytosolic cuproenzyme SOD1 by CCS. Moreover, it has been found that targeting CCS could yield neuroprotective effects after ischemic damage in the hippocampus of gerbils. In addition, CCS depletion was shown to impair angiogenesis and induce the progression of multiple types of CVDs (Manzl et al., 2004; Chen et al., 2023). Further investigations of small molecule inhibitors of copper chaperone proteins are required to protect against CVDs.

#### Copper ionophore

Copper ionophores are small molecules that transport copper into cells and are used in therapeutic methods targeting copper deficiency (Su et al., 2018). Although Elesclomol is widely used in clinical settings to target cancer cells, the mechanism behind its selectivity is still unknown (Li et al., 2022b). Conventional copper ion carriers have limitations in terms of versatility and lack of targeted delivery. Copper transport cannot be precisely regulated since excessive copper supplementation and Fenton-like chemotaxis may lead to oxidative damage (Yuan et al., 2017). Inappropriate copper transport may lead to tissue defects. If copper is delivered to nonspecific tissues, it can accumulate and cause oxidative damage. As a result, the concept of targeted iron carrier-based metal supplements (TIMS) has emerged, aiming to transport metals specifically to a particular site within an organism (Su et al., 2019). N-acetylgalactosamine-functionalized ions were introduced and produced, which are more effective in transporting copper to the liver than other nontargeted Cu (gtsm) ion carriers (Su et al., 2018). This approach sheds light on applying metals in the treatment of CVDs.

A possible alternative to address copper deficiency is the implementation of a nanodrug delivery system. A multifunctional nanocomposite has been recently developed that combines CuS photothermal therapy with antiatherosclerotic chemotherapy and can release drugs effectively at the site of the inflammatory atherosclerotic environment. This delivery system enables the targeted transport of treatments for atherosclerosis. Emphasis should be placed on enhancing specificity and targeting capabilities to prevent unwanted delivery of copper to nonspecific tissues for the future development of selective copper ionophores.

### Outlook

Copper is a double-edged sword in cells; although it is an essential cofactor for many enzymes, excessive amounts can cause oxidative stress and cell death. Given the significance of cuproptosis, other disorders in metal ion metabolism are also of research interest for exploring disease mechanisms and developing therapeutic targets. Targeting copper ion metabolism has emerged as a potentially effective means of treating tumors in tumor research. The lethal effect of copper on cells at a certain concentration threshold has long been applied in the clinic, such as disulfiram and copper ion therapy for tumors, which is based on the phenomenon of cuproptosis. However, understanding the specific mechanism of copper death and achieving more precise targeted therapy remains a focus in cuproptosis research.

There is an increasing consensus that copper does not significantly affect mitochondrial respiration but exhibits potent toxicity in cells with high metabolic activity and respiration due to its effect on fatty acylase in the tricarboxylic acid cycle, which is abundant in these cells. Targeting cancer cells selectively with copper ion carriers through drug delivery systems (DDS) is a promising approach to overcoming dose-related limitations. Nonetheless, whether this approach could be used to selectively deliver drugs to hypertrophic myocardium requires further investigation.

A recent example shows that a copper death-inducing nanoparticle drug, NP@ESCu, can induce cuproptosis in cancer cells and reprogram the immunosuppressive tumor microenvironment (Guo et al., 2023). When combined with an immune checkpoint inhibitor (αPD-L1), it can enhance cancer treatment and significantly inhibit tumor growth and has broad clinical application prospects (Guo et al., 2023). Elesclomol is a potent copper ionic carrier that promotes cuproptosis. Its special function of transporting copper ions to cellular mitochondria indicates its therapeutic potential in treating rare copper deficiency diseases, such as Menkes disease (Zheng et al., 2022). However, the potential role of related inhibitors in CVDs and whether they can be used as potential therapeutic agents still need to be further studied.

Although clinical trials of ilimol as a cancer treatment failed, subsequent analyses indicated that the molecule might benefit patients whose tumors rely on mitochondria for energy. Markers of copper-induced cell death have been identified, suggesting that ilimol can potentially treat various diseases susceptible to copper death, including cancers that express FDX1. However, whether copper ion carrier drugs such as ilimol can treat heart-related diseases remains unclear, emphasizing the need for future studies.

Indeed, the dysregulation of copper metabolism can also cause changes in the cardiac expression of genes (Wei et al., 2012). Supplementing copper can reverse several of the negative effects of copper deficiency in the heart. Since changes in copper homeostasis are associated with multiple disease conditions, further research is warranted to determine how copper imbalance leads to cellular damage. This information could be valuable in designing treatments to prevent or slow disease progression.
